# New Targets in Heart Failure Drug Therapy

**DOI:** 10.3389/fcvm.2021.665797

**Published:** 2021-05-05

**Authors:** Michele Correale, Lucia Tricarico, Martino Fortunato, Pietro Mazzeo, Savina Nodari, Matteo Di Biase, Natale Daniele Brunetti

**Affiliations:** ^1^Department of Cardiology, Policlinico Riuniti University Hospital, Foggia, Italy; ^2^Department of Medical and Surgical Sciences, University of Foggia, Foggia, Italy; ^3^Cardiology Section, Department of Medical and Surgical Specialties, Radiological Sciences and Public Health, University of Brescia, Brescia, Italy

**Keywords:** drug therapy, therapy targets, heart failure, inflammation, micro-circulation, interstitium

## Abstract

Despite recent advances in chronic heart failure management (either pharmacological or non-pharmacological), the prognosis of heart failure (HF) patients remains poor. This poor prognosis emphasizes the need for developing novel pathways for testing new HF drugs, beyond neurohumoral and hemodynamic modulation approaches. The development of new drugs for HF therapy must thus necessarily focus on novel approaches such as the direct effect on cardiomyocytes, coronary microcirculation, and myocardial interstitium. This review summarizes principal evidence on new possible pharmacological targets for the treatment of HF patients, mainly focusing on microcirculation, cardiomyocyte, and anti-inflammatory therapy.

## Introduction

Despite significant improvements in cardiovascular (CV) mortality over the last decades, CV disease is the main reason for death in several countries. CV therapies improved the survival of patients with CV disease, but, at the same time, increased the number of subjects affected by chronic CV conditions such as heart failure (HF).

**Figure 1:**
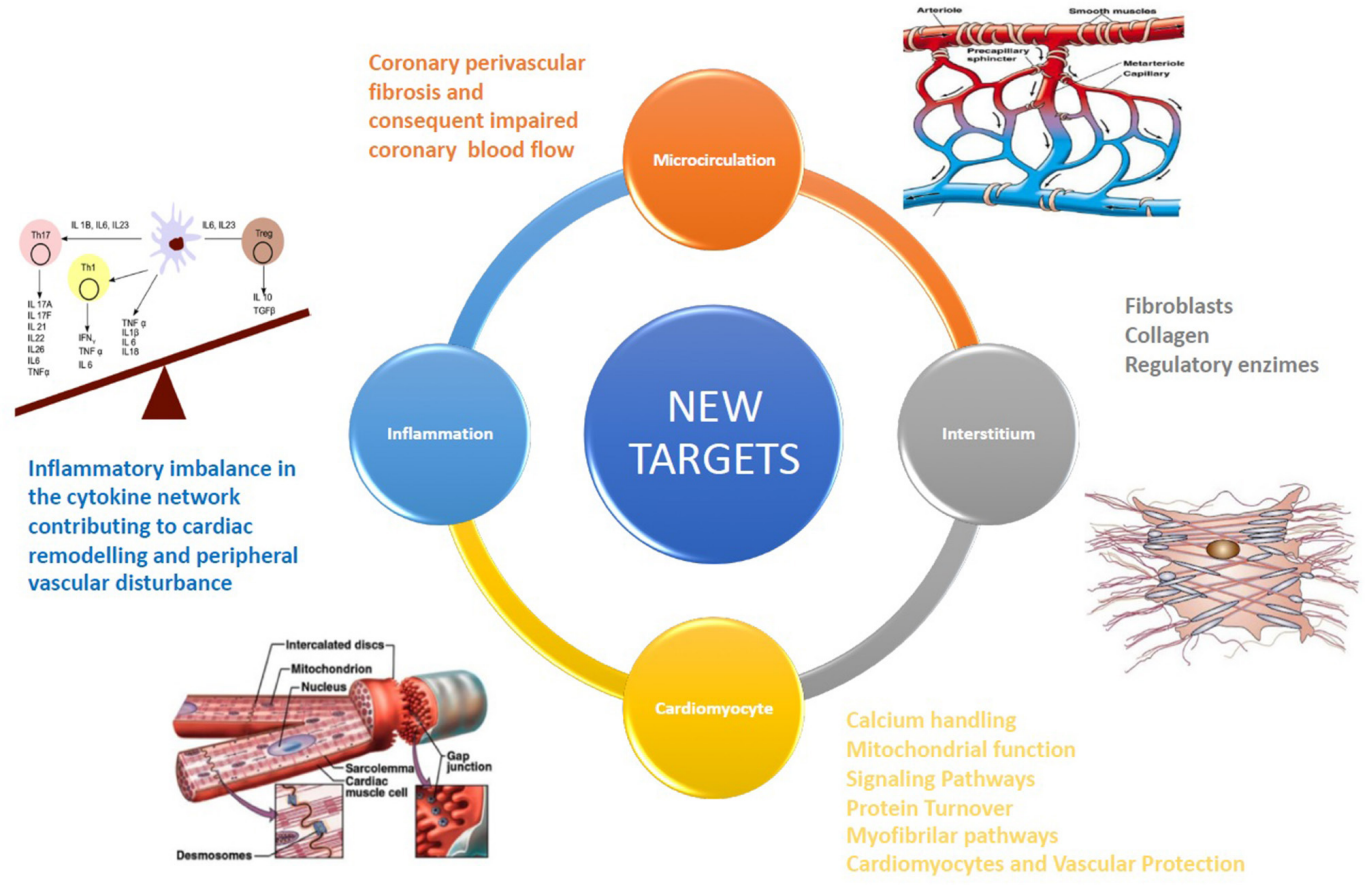
The development of new drugs for HF therapy must be necessarily focused on additional targets, such as cardiomyocytes, coronary microcirculation, and myocardial interstitium.

Several clinical randomized studies assessed the efficacy of drug therapies in patients with HF. Residual CV mortality, however, remains considerable (1), potentially because novel therapeutic options are too often pursued with an “old” approach, with non-targeted and non-personalized therapies, not based on individual pathophysiology. Each patient affected by HF, instead, may be characterized by different etiologies, clinical characteristics, and comorbidities, each one to be possibly considered (2). Benefits from drugs based on neurohumoral and hemodynamic modulation (3) quickly reach a therapeutic plateau, with very limited possible additional benefit from incremental doses or additional drugs based on the same pathway. Recently, a novel paradigm has been reported in HF with preserved ejection fraction (HFpEF). Some comorbidities (obesity, diabetes mellitus, and chronic obstructive pulmonary disease) drive myocardial dysfunction through coronary microvascular endothelial inflammation in HFpEF. These comorbidities lead to a systemic proinflammatory state causing coronary microvascular endothelial inflammation (4). The development of new drugs for HF therapy must be necessarily focused on additional targets (anatomical or structural as cardiomyocyte and myocardial interstitium, and physiological or functional as microcirculation and inflammation) [(5); Figure 1] above all for HFpEF. Detailed knowledge of the interstitium and of cardiomyocyte biology becomes therefore essential; we thus briefly report principal evidence on new possible pharmacological targets for the treatment of HF patients (Table 1), mainly focusing on microcirculation, cardiomyocyte, and anti-inflammatory therapy.

**Table 1:** New possible pharmacological targets and promising drugs for the treatment of HF patients.

**Area target**	**Targets**	**New promising drugs**	**HF form**	**References**
Microcirculation	Targeting EC metabolism Targeting EC nutrient transport	Only translation drugs	HFpEF	(6)
Interstitium	Myocardial interstitial fibrosis Chymase	Sacubitril/valsartan; Empagliflozin Fulacimstat	HFrEF HFpEF	(7) (8) (9)
Cardiomyocyte (calcium handling)	SERCA2a	Istaroxime		(10)
Cardiomyocyte (nitroxyl donors)	SERCA2a	BMS-986231	AHF-HFrEF	(11, 12)
Mitochondria	Partial adenosine A1 receptor	Neladenoson	HFrEF; HFpEF	(13, 14)
	Cytochrome C	Elamipretide	HFrEF	(15)
Signaling pathways	Soluble guanylate cyclase (sGC)	Vericiguat	HFrEF; HFpEF	(16–18)
	Arginine vasopressin signaling	Pecavaptan	HFrEF; HFpEF	(19)
Myofibrillar pathway	Myosin	Omecamtiv mecarbil	HFrEF	(20–23)
Inflammation	Interleukin-1 receptor	Canakinumab	HFrEF	(24)
	Interleukin-1 receptor	Anakinra	HFrEF	(25)

## Microcirculation

A functional and “healthy” coronary microcirculation is essential for myocardial activity and effectiveness. It plays a pivotal role in the regulation of coronary blood flow in response to cardiac oxygen requirements. Impairment of this mechanism is defined as coronary microvascular dysfunction (CMD) (26). Microcirculatory anomalies can be often found in HF patients (27) and parallel disease progression in ischemic HF (28). CMD is associated with the development of HFpEF. In fact, decreased bio-accessibility of nitric oxide (NO) in endothelial dysfunction plays an important role in HF (29), mainly in HF with (HFpEF) (30). In HFpEF models, coronary microvascular endothelial inflammation reduces nitric oxide bioavailability, cyclic guanosine monophosphate content, and protein kinase G (PKG) activity in adjacent cardiomyocytes (31).

Recent reports suggest that the microcirculation has additional roles in supporting a healthy microenvironment (6). Experimental models have showed that restoring a healthy microcirculation and endothelium could be a possible therapeutic approach to treat HF.

Coronary perivascular fibrosis and the consequently impaired coronary blood flow may represent a new therapeutic target to improve coronary microcirculation (32).

Finally, there is emerging evidence about new translational drugs on the microcirculation (including growth factors and non-coding RNA therapeutics, as well as the targeting of metabolites or metabolic signaling) (6).

Forthcoming trials would better assess coronary microcirculation by cardiac magnetic resonance imaging (cMRI) (33, 34).

## Interstitium

The knowledge of interstitium biology is essential for the development of new drugs for HF, providing several potential therapeutic targets in the case of HF: fibroblasts, collagen, and regulatory enzymes regulating collagen synthesis. Myocardial interstitium is not an inert scaffold, but rather an elaborate and active micro-habitat within the myocardium (35). HF fibrotic changes in the interstitium and near capillaries are featured by extracellular matrix (ECM) expansion and myofibroblast secretion of type I collagen. The level of collagen type I crosslinking is related to increased filling pressures in HF patients (36). A new cMRI technique, the T1 mapping (measures the extracellular volume fraction, ECV in human myocardium) permits the distinction of different components of interstitium (cardiomyocytes and connective tissue) and a more precise definition of myocardial fibrosis (37). ECV can be used as a tool in phase II trials to assess the efficacy of novel anti-myocardial fibrosis therapeutics (38).

Myocardial interstitial fibrosis (MIF) is very common in patients with HFpEF and with HFrEF. It is related to cardiac function impairment and poor outcome. It is determined by the changes in the quantity and quality of collagen fibers and in the ECM (39).

Pharmacological drugs formerly utilized with demonstrated safety may also be interesting for the treatment of MIF by means of new mechanisms. The sacubitril/valsartan and the sodium-glucose cotransporter 2 (SGLT2) inhibitor empagliflozin decreased MIF in HF mice with diabetes and improved LV function (7, 8). However, sacubitril/valsartan, in a recent phase III clinical research trial in patients with HFpEF, showed just a marginal reduction of the primary composite endpoint of total hospitalizations for HF and death from CV origin (40). Currently, SGLT2 inhibitors are still under evaluation to determine if they can effectively reduce MIF in humans.

An antifibrotic action was also demonstrated by pirfenidone and tranilast, through the inhibition TGF-β signaling. Prolonged usage of such drugs, however, may cause hepatic toxicity and may culminate in liver failure, therefore further studies are necessary to search for new effective TGF-β pathway targets, but safely too, for MIF reduction (41).

Chymase is a chymotrypsin-like serine protease that is secreted from activated mast cells and other cells, such as cardiomyocytes in the case of tissue damages (42, 43). Chymase is produced after secretion and produces or activates locally profibrotic factors, such as angiotensin (Ang) II, transforming growth factor (TGF) β, and matrix metalloproteinases that take part in adverse remodeling post-MI (44). For these reasons it can be a potential new target for post-MI therapy. Fulacimstat is an orally existing chymase inhibitor which has a multi-functional anti-remodeling effect that reduces LV disfunction after myocardial infarction (9).

## Cardiomyocyte

### Calcium Handling

Abnormal handling of intracellular Ca^2+^ in cardiomyocytes plays an important role in impaired cardiac contractility of HF. Ca^2+^ homeostasis is maintained in cardiomyocytes by ryanodine receptor and Ca-calmodulin kinase IIdelta (CaMKIIdelta), acting separately to manage cardiac Ca^2+^ handling, which was impaired in cardiac dysfunction (45). Until now there are only translational drugs. However, istaroxime may be a promising drug in the future. Istaroxime is a molecule with a luso-inotropic effect in HF, across the stimulation of SERCA2a ATPase activity and the augmentation of Ca^2+^ uptake into the sarcoplasmatic reticulum (SR) by mitigating the phospholamban inhibitive effect on SERCA2a in a cAMP/PKA independent way (46). In patients affected by HFrEF during hospitalization for AHF, a 24-h infusion of istaroxime improved the parameters of diastolic and systolic cardiac function without major cardiac adverse effects (10).

SERCA2a is in charge of sequestrating cytosolic Ca^2+^ back into the sarcoplasmic reticulum, enabling an effective uncoupling of actin-myosin and upcoming ventricular relaxation. Previous studies have proved that the expression of SERCA2a is downregulated in CHF, which later leads to serious systolic and diastolic function impairment.

JTV519 (K201) consolidates the closed state of type-2 ryanodine receptor (RyR2) by improving its affinity for FKBP12.6, which inhibits the leakage of Ca^2+^. K201 prevents spontaneous diastolic Ca^2+^ issued during Ca^2+^ overload by a double inhibitory effect on SR Ca^2+^-ATPase (SERCA2a) and RyR2, with no antiarrhythmic impact (47).

Another potential target for new HF drugs may be Na^+^-H^+^ exchanger-1 (NHE-1). Treatment with cariporide, an NHE-1 inhibitor, induced the reversal of hypertrophy and HF and the attenuation of apoptosis in cardiomyocytes (48), with some antiarrhythmic effects (49).

Nitroxyl (HNO) donors are compounds which improve cardiomyocyte function by direct improvement of SR Ca^2+^ cycling. BMS-986231 demonstrated a favorable safety and hemodynamic profile in patients hospitalized with advanced HF (11). An ongoing phase II randomized placebo-controlled clinical trial (StandUP-AHF) was designed to provide evidence of tolerability and efficacy of BMS-986231 in patients with HFrEF (12).

Nucleotide-binding oligomerization domain-containing protein 1 (NOD1) is a recent accredited innate immune sensor implicated in CV diseases. The NOD1 pathway was over-expressed in human and murine failing myocardia. Val-Blasco et al. (50) demonstrated that NOD1 may modulate intracellular Ca^2+^ mishandling in HF, appearing as a novel target for HF therapy.

### Mitochondrial Function

In HF patients, several mitochondrial anomalies have been found. Impaired mitochondrial electron transport chain activity, enhanced reactive oxygen species (ROS) formation, changed metabolic substrate usage, abnormal mitochondrial dynamics, and modified ion homeostasis are currently being studied (51).

Neladenoson and elamipretide are the most promising drugs affecting mitochondrial function. Neladenoson, the novel partial adenosine A1 agonist, seems to be harmless, with no atrioventricular conduction or neurological side effects in HFrEF patients (13). In particular, the treatment was not related to dose-dependent favorable effects on cardiac function or clinical outcome but was linked with a dose-dependent renal function reduction (14) and no significant change in exercise capacity in patients with HFpEF (52).

Elamipretide is a water-soluble tetrapeptide capable of entering the inner mitochondrial membrane and joining with the phospholipid cardiolipin (53). This association leads to a stabilized cytochrome c conformation in order to promote effective electron transport in mitochondria and improve oxidative phosphorylation (54). LVEF, LV end diastolic pressure, cardiac hypertrophy, myocardial fibrosis, and cardiac ATP synthesis improvement in animal models and humans in treatment with elamipretide was demonstrated (55–57). On the other hand, in the PROGRESS-HF (phase 2) trial, elamipretide did not show a statistically significant change of LV end systolic volume in stable HFrEF patients compared with placebo after 4 weeks (15). More clinical studies of elamipretide in other HF phenotypes are required to assess its potential role in HF. At the same time longer term trials in HFrEF may be necessary too.

Capadenoson, a partial adenosine A1R agonist through inhibition of mPTP opening, might affect mitochondrial function (58). Another potential agonist (VCP746), an atypical A1AR agonist, has been investigated in order to develop ligands that promote A1AR cytoprotection in the absence of adverse hemodynamic effects (59).

Inhibition of mitochondria permeability transition pore (mPTP), in failing cardiomyocytes with cyclosporine A, maintained the expression of cytochrome c oxidase, increasing mitochondrial cytochrome c oxidase-dependent respiration and ATP synthesis (60). Sevoflurane, a volatile anesthetic, increases the threshold of calcium-induced mPTP opening (61).

Mitochondria are an important ROS source, and progress has been achieved to understand the way the matching of energy supply and demand across calcium handling might impact mitochondrial ROS development and removal. In mitochondria, oxidative stress generates a switch toward fission that leads to mitochondrial fragmentation and cell death. Doxycycline defends mitochondria against oxidative stress, and regulates the mitochondrial function causing a change of equilibrium toward fusion, so it might be an innovative therapeutic approach for HF (62).

Ca^2+^/calmodulin-dependent protein kinase II (CaMKII) has a crucial role in the progression of HF and in the generation of myocardial mitochondrial injury. Inhibition of CaMKII avoids Ca^2+^ intake toward mitochondria and decreases destruction of these organelles. Therefore, rising evidence supports the targeting of CaMKII and the mPTP as a way to preclude tissue damage. H_2_S-induced inactivation of CaMKII also may allow for a new therapeutic approach for CV diseases (63).

This approach might provide new possibilities to improve mitochondrial function in HF by fixing cytosolic and mitochondrial ion transporters. So, very specific molecules such as the preclinical CGP-37157, or ranolazine and empagliflozin (64), may be effective. Ranolazine decreases pressure overload-induced cardiac hypertrophy and improves cardiac function by preserving Na^+^ and Ca^2+^ handling (65). Empagliflozin decreases CaMKII activity (66). However, mitochondrial biogenesis, the production of ROS, and maintenance of cellular iron homeostasis need further studies to confirm their possible role for novel therapies in HF (67).

The GLP-1 receptor agonist liraglutide might have a therapeutic role in the modulation of cardiac inflammation. Liraglutide improved IL-1β-induced cellular ROS production and NADPH oxidase (NOX)-4 expression. Furthermore, it preserved cardiomyocytes from IL-1β-induced reduced mitochondrial membrane potential and decreased ATP production (68).

Melenovsky et al. demonstrated that, in the presence of profoundly impaired mitochondrial function in HF patients, myocardial iron deficiency may aggravate mitochondrial metabolism and ROS handling. So, the restoration of good levels of myocardial iron might help to ameliorate the bioenergetics of HF (69).

Acetylation of mitochondrial proteins was demonstrated to play an active role in the pathogenesis of cardiac diseases. Proteins are deacetylated by NAD^+^-dependent deacetylases so-called sirtuins (SIRTs). SIRT3, with the regulation of the activity of enzymes involved in ATP production in the mitochondria, might keep the equilibrium between cardiac function and energy consumption. Downregulation of SIRT3 may lead to the unbalance of mitochondrial bioenergetics related to impaired mitochondrial function. Therefore, the preservation of SIRT3 activity may be a possible approach to avoid pathological consequences of cardiopathies (70).

Myocytes have control mechanisms to maintain functional mitochondria with the removal of compromised mitochondria through specialized autophagy. Huang et al. observed that IGF-IIR signaling impaired expression and circulation of dynamin-related protein (Drp1) and mitofusin (Mfn2) (71). IGF-IIR activates JNK-mediated Bcl-2 phosphorylation to stimulate ULK1/Beclin 1-dependent autophagosome development. Unreasonable mitochondrial fission by Drp1 improved the Rab9-dependent autophagosome identification and swallowing of damaged mitochondria and eventually reduced cardiomyocyte viability. These results showed the link between Rab9-dependent autophagosomes and mitochondrial fission in cardiomyocytes, which gives another potential therapeutic approach.

### Signaling Pathways

Reduced NO bioavailability leads to a relative sGC deficiency and a reduction in cGMP synthesis, essential for normal cardiac and vascular function (72–74). Cyclic GMP ameliorates endothelial function and reduces cardiac fibrosis. Direct NO-independent sGC stimulation might provide a new chance to address the relative cGMP deficit in HF. Vericiguat is a novel soluble guanylate cyclase (sGC) stimulator, tested in the SOCRATES PRESERVED study (16), REDUCED study (17), and recently also in the VICTORIA trial (18). It avoids that nitric oxide step and directly binds to induce the sGC. This promising drug raises solid cGMP production and produces the subsequent beneficial effects on the CV system. The VICTORIA trial showed that patients taking vericiguat were 10% less likely to have the primary outcome, a composite of CV death or first hospitalization for HF, than those taking placebo.

Increased arginine vasopressin signaling in either or both the V1a and V2 receptors could contribute to the development of HF. V1a activation could cause vasoconstriction, cardiac hypertrophy, and fibrosis (75) as intracellular signaling pathways are closely related to those for angiotensin II. Renal tubular V2 activation could cause water retention and hyponatremia. A novel dual-balanced vasopressin antagonist (pecavaptan) may lead to decongestion (via V2) and protect heart and vessels (via V1a), improving outcomes. It was shown to be superior to tolvaptan in preclinical models (76). This is the rationale for an ongoing trial (AVANTI) (19) which will enroll HFpEF and HFrEF patients with incomplete decongestion despite standard therapy at day 3–7 after index hospitalization.

All other options are translational drugs. Some receptor-mediated signaling pathways (natriuretic peptides, mediators of glycogen synthase kinase 3 and ERK1/2 pathways, beta adrenergic receptor subtypes, relaxin receptor signaling, TNF/TNF receptor family, TWEAK/Fn14 axis, and micro-RNAs) may represent targets for emerging therapies in HF treatment (77). miRNAs are endogenous non-coding single-stranded RNAs that control gene expression and regulate adaptive and maladaptive cardiac remodeling (78, 79). By blocking the ERK1/2 pathway it is possible to prevent progression of CHF by reducing fibrosis, inflammation, and apoptosis in the myocardium (80).

Recently, more attention has been given to signaling pathways linking cysteine cathepsins. They play a role in synthesis and degradation of the ECM, affecting ventricular remodeling and contractile capacity (81).

The potential therapeutic strategy targeting protein phosphatase 1 (I-1) by restoring the balance of cardiac protein phosphorylation needs further study in order to prove its real clinical benefit (82).

G-protein-coupled receptor (GPCR) kinase-2 (GRK2) is a regulator of GPCRs, in particular beta adrenergic receptors, and has a crucial role in the development and advance of CV disease, like HF. GRK2 inhibition has been examined in several models (83); in a rat model, inhibition by a cyclic peptide C7, improved mitochondrial activity, and improved the contractility (84).

### Myofibrillar Pathways

Myosin activators, such as omecamtiv mecarbil and danicamtiv, facilitate the rate-limiting step of the myosin enzymatic cycle and switch the cycle for the benefit of the force-producing state. Myosin activators improve cardiac contractility by increasing the conversion of the actin–myosin complex from a weak to a strong bond, with no variation of intracellular Ca^2+^ homeostasis, increasing LV systolic function, and without improvement of energy request or arrhythmogenesis (85–87). Omecamtiv mecarbil ameliorates left ventricle systolic ejection time, SV, and CO, without side effects from the inotropic drugs; its bioavailability is high and the drug is safe in HFrEF (88). As shown in the ATOMIC-AHF trial in symptomatic CHF and LVEF lower than 40%, the drug was not associated with improved dyspnea but it did increase systolic EF, decrease the LV end-systolic diameter, and was well-tolerated (20, 21). It also significantly decreased NT-pro-BNP compared with placebo (22); it was also safe for acute HF.

Its effects on CV outcomes were tested in the GALACTIC-HF trial (23). Among patients with HFrEF, those who received this drug revealed a significant relative risk reduction in a composite of a HF event or CV death, compared with placebo. A phase III study (METEORIC-HF) is currently ongoing to assess its effects on exercise capacity^1^. This new drug seems to be a promising new approach for patients affected by HFrEF.

The giant protein titin is anchored at the Z-disc; changes in titin stiffness occur in HF through a switch in the expression fraction of the two major titin isoforms in cardiac sarcomeres, N2BA (compliant) and N2B (stiffer). In HF patients, increased passive stiffness can be detected because of a titin phosphorylation deficit (89), thus representing another potential drug target in HF.

## Anti-inflammatory Effects of Drugs

To the best of our knowledge, there are not sufficiently valid data (anti-TNF-α drugs have not shown benefits in CHF) about anti-inflammatory drugs in HF. Previous studies showed that inflammatory mediators may be relevant in the CHF, contributing to adverse remodeling and peripheral vascular anomalies. Inflammatory mechanisms are also hypothesized in HFpEF, with pro-inflammatory/pro-fibrotic or immunological alterations (90).

Previous research showed increased values of inflammatory cytokines such as TNF-α, IL-1β, and IL-6 in HF. In HF patients, this improvement in inflammatory mediators does not appear to be joined by an equivalent increase in anti-inflammatory cytokines such as IL-10 and TGF-β; thus resulting in an inflammatory imbalance in the cytokine system (91). Interleukin-1β (IL-1β) is known to depress cardiac function. A small secondary analysis of the CANTOS (Canakinumab Anti-inflammatory Thrombosis Outcome Study) trial (24) confirmed IL-1 as a potential therapeutic target in HF. Instead, in patients with LVEF <50% and administration of anakinra (IL-1 receptor antagonist) was only associated with exercise capacity improvement after 12 weeks of therapy (25).

Recently in some drugs already used in HF therapy, such as glycosides (92) and ivabradine (93), anti-inflammatory effects have been demonstrated.

Others authors have proved the possible advantage of the combination of methotrexate with conventional therapy, through the interaction between the activated immune and inflammatory mediator's system (94).

Pentoxyfylline, intravenous immunoglobulin, thalidomide, and statins, have demonstrated encouraging results in smaller studies (95), although these have not been verified in multicenter randomized studies with relevant endpoints (96–101). In a meta-analysis, lipophilic statins were superior to hydrophilic statins in terms of follow-up LVEF, BNP, C-reactive protein, and IL-6 in HF (102).

Some recently discovered therapeutic targets (peroxisome proliferator-activated receptor gamma activators, Rho-kinase, p38 mitogen-activated protein kinase, nuclear transcription factor NF-kappaB) modulating parasympathetic tone, macrophage inhibitors, and chemokine receptor antagonists, have already been tested.

Oleuropein mitigates the progression of HF, presumably by anti-oxidative and anti-inflammatory effects (103). Also oral taurine supplementation in HF with LVEF <50% has anti-atherogenic and anti-inflammatory effects (104).

Another possible strategy can be represented by resveratrol. The resveratrol treatment decreased the galectin-3 level, which is secreted by macrophages, and plays a role in mediating cardiac fibrosis and inflammation. This leads to the reduction of IL-1 and IL-6 levels. The reduced activity of leukocytes may be a significant effect of resveratrol, and it can lead to heart function improvement in HFrEF (105).

## Future Developments

The most encouraging results for near future developments are given by gliflozins.

Encouraging results in type 2 diabetic patients with prior CV events from the EMPAREG-HF study (empagliflozin) (106), from the CANVAS program (canagliflozin) (107, 108), and from the DECLARE-TIMI 58 trial (dapagliflozin) (109) induced researchers to test SGLT2i in people with HF regardless of the presence of diabetes mellitus. Results from the DAPA-HF trial (110) and from the EMPEROR-Reduced trial (111) in both diabetics and non-diabetics showed an important reduction in CV death and HF hospitalization. The mechanisms underlying the cardiac effects of gliflozins have not yet been fully defined, although possible effects of these drugs on cardiomyocytes could be supposed. Ongoing clinical trials will clarify the effects and mechanisms of gliflozins in HFrEF and HFpEF patients with and without T2DM (112, 113). New results have been derived from the studies on endothelial function in CHF (114), especially interesting results have been obtained in HFpEF in treatment with empagliflozin (115, 116).

Other possible approaches for the future include RyR2 and its associated accessory proteins, which might be potential new drug targets. RyR2 placed on the sarcoplasmic reticulum produces systolic Ca^2+^ transients within cardiomyocytes. Appropriate functioning of RyR2 is therefore pivotal to the timing and force produced by cardiomyocytes. Impaired intracellular Ca^2+^ handing secondary to impaired function of RyR2 may be related to HF (117).

Normal cardiac Ca^2+^ handling is also due to striated muscle preferably expressed protein kinase (SPEG), a member of the myosin light chain kinase family. SPEG has been causally linked to HF and atrial fibrillation, so it can be taken into consideration (118).

Gene therapy is still far from being clinically applicable (119). The gene cysteine-rich secretory protein LCCL domain containing 1 (CRISPLD1) is overexpressed in HF. The downregulation of some signaling pathways upon CRISPLD1-KO implicates a role in adverse remodeling. These discoveries offer novel candidate genes with encouraging potential roles for therapeutic interventions (120).

The cardiac bridging integrator 1 gene (cBIN1) therapy stabilizes the subcellular membrane within cardiomyocytes, preserving intracellular distribution of calcium (121), and potentially could be a new way forward to find new drugs.

## Conclusions

Possible new HF drugs, above all for HFpEF, might target myocytes, mitochondria, microvascular circulation, and interstitium. Further studies are needed to transfer evidence derived from pre-clinical evidence to real-life with new possible therapeutic approaches aimed at new therapeutic targets in HF.

## Author Contributions

MC and SN: substantial conception. LT, MF, and PM: acquisition of data. MD: drafting of the article for important intellectual content. NB: revising the article for final approval of the submitted paper. All authors contributed to the article and approved the submitted version.

## Conflict of Interest

The authors declare that the research was conducted in the absence of any commercial or financial relationships that could be construed as a potential conflict of interest.

## Footnotes

^1^ClinicalTrials.gov Identifier: NCT03759392.
